# Data on CXC chemokine ligand 10(CXCL10) expression and activation in red sea bream during bacterial and viral infection

**DOI:** 10.1016/j.dib.2019.103943

**Published:** 2019-04-23

**Authors:** Won-Sik Woo, Min Soo Joo, Jee Youn Hwang, Mun-Gyeong Kwon, Jung Soo Seo, Seong Don Hwang, Bo-Yeong Jee, Mu-Chan Kim, Chan-Il Park

**Affiliations:** a Institute of Marine Industry, College of Marine Science, Gyeongsang National University, 455, Tongyeong 650-160, Republic of Korea; b Aquatic Animal Disease Control Center, National Institute of Fisheries Science (NIFS), 216 Gijanghaean-ro, Gijang-eup, Gijang-gun, Busan 46083, Republic of Korea

## Abstract

CXCL10 plays an important role in angiogenesis and inhibits the differentiation of endothelial cells into capillaries. It also plays an important role in the generation and transmission of effector T cell responses and the recruitment of T cells to inflammatory sites. In this article, we constructed cDNAs to identify and analyse the CXCL10 domain, and performed multiple alignments and a phylogenetic analysis to determine homology with other animals. Real-time PCR was performed to confirm construction and expression after bacterial and viral infection.

**Table:** Specifications Table

Subject area	Aquaculture Biology
More specific subject area	Molecular Biology
Type of data	Table, figure
How data was acquired	The nucleotide sequence was confirmed using Genetyx 7.0. Phylogenetic classification was performed using the Mega 5.0 program, and the expression was confirmed by real–time PCR (TaKaRa Thermal Cycler Dice Real Time System Single).
Data format	Analysed, Realtime PCR
Experimental factors	Full-length cDNA from CXCL10 was obtained from next generation sequencing (NGS) analysis using the liver of red sea bream stimulated with *Streptococcus iniae* and Red Seabream Iridovirus (RSIV).
Experimental features	Primers were designed using Primer3 (version 0.4.0) and the assay precision was determined by real–time PCR.
Data source location	National Institute of Fisheries Science, Busan, South Korea
Data accessibility	Data are with this article
Related research article

**Table:** 

**Value of the Data** • The immune related gene of red seabream can be used as the basis for the immune system of red seabream. • The sequence analysis confirms homology with other species and it may be useful for subsequent immune studies in other species. • The real–time PCR data can be used as baseline data after long-term infectious pathogen infection and compared with other species. • The data generated in this study will contribute to identifying a biomarker that can diagnose the pathogenicity in fish at an early stage.

## 1 Data

**Fig. 1:**
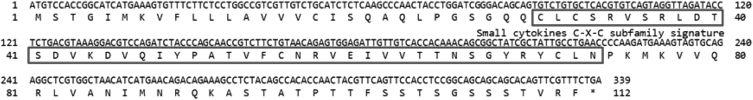
cDNA and deduced amino acid sequence of the CXCL10. The CXCL10 domain is indicated by the box.

**Fig. 2:**
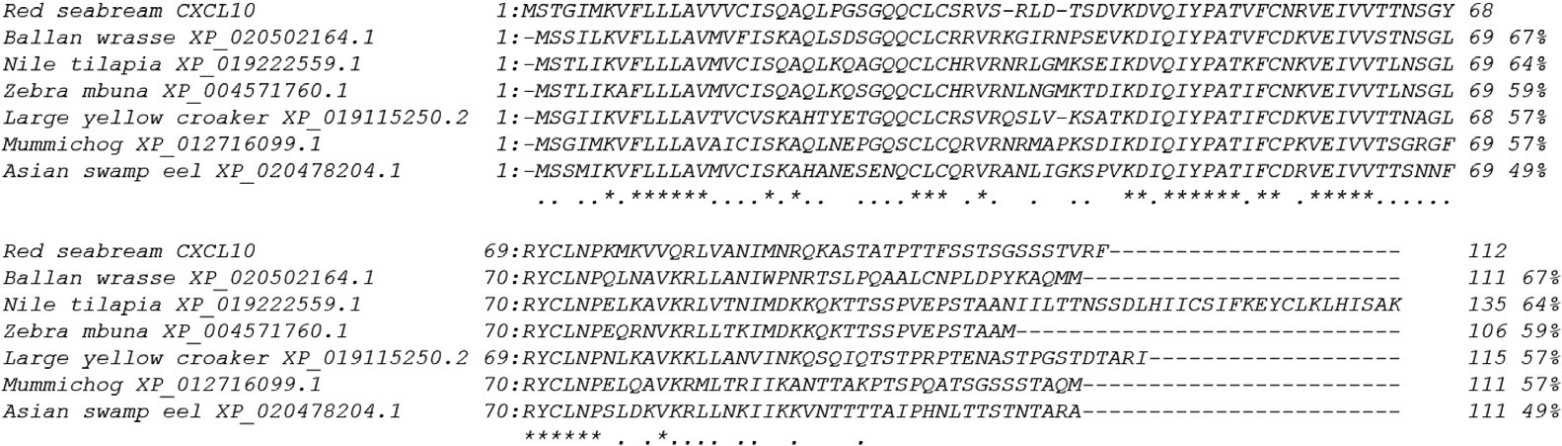
Multiple alignments of CXCL10 with the AMPs amino acid sequences of the other fish. The NCBI accession numbers for dicentracin are as follows: Ballan wrasse XP_020502164.1; Nile tilapia XP_019222559.1; Zebra mbuna XP_004571760.1; Large yellow croaker XP_019115250.2; Mummichog XP_012716099.1; Asian swamp eel XP_020478204.1.

**Fig. 3:**
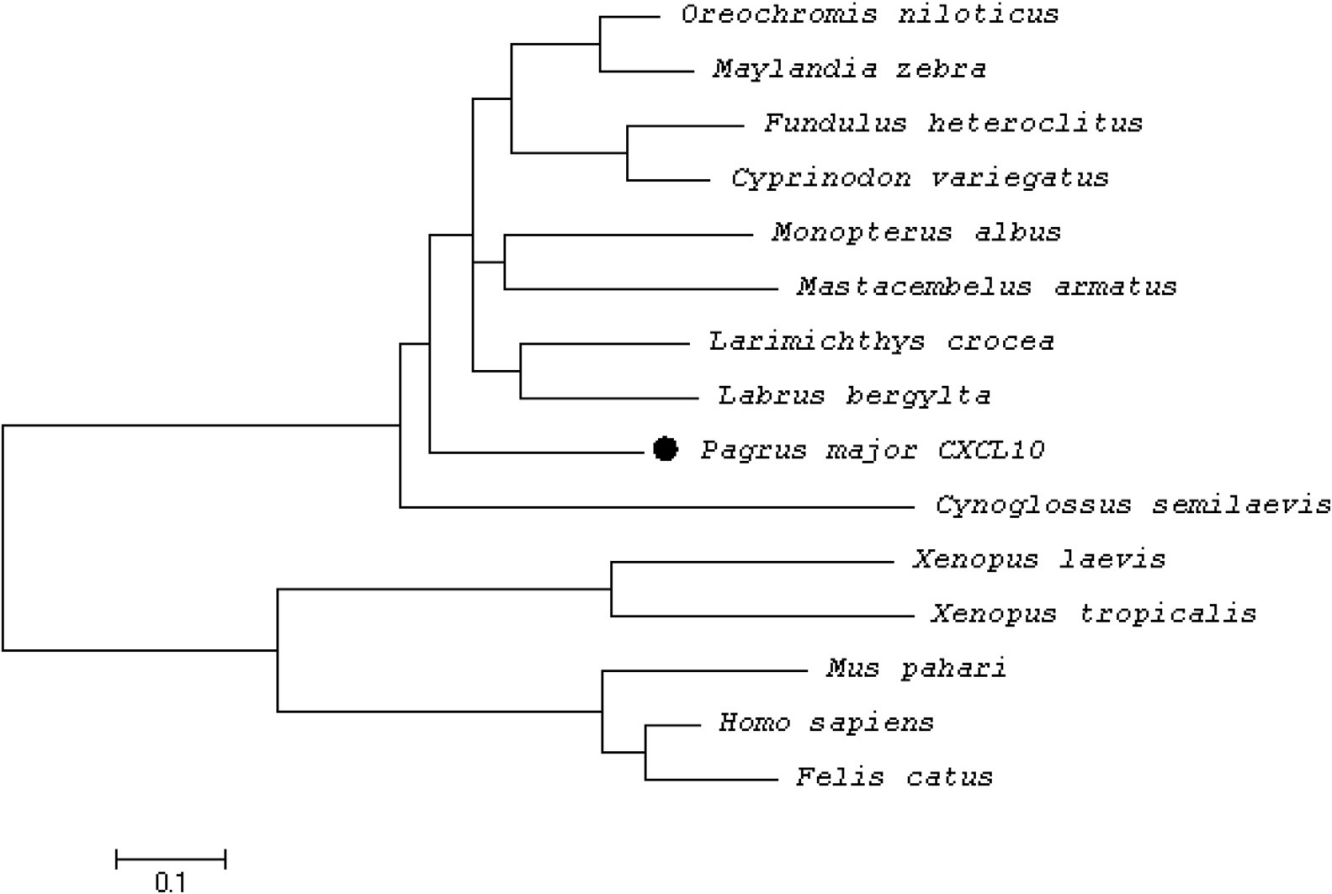
Phylogenetic analysis of the deduced CXCL10 amino acid sequences in fish and other species. The phylogenetic tree was constructed using the neighbour-joining method within MEGA 4 software. Bootstrap sampling was performed with 2000 replicates. The scale bar is equal to 0.1 changes per amino acid position.

**Fig. 4:**
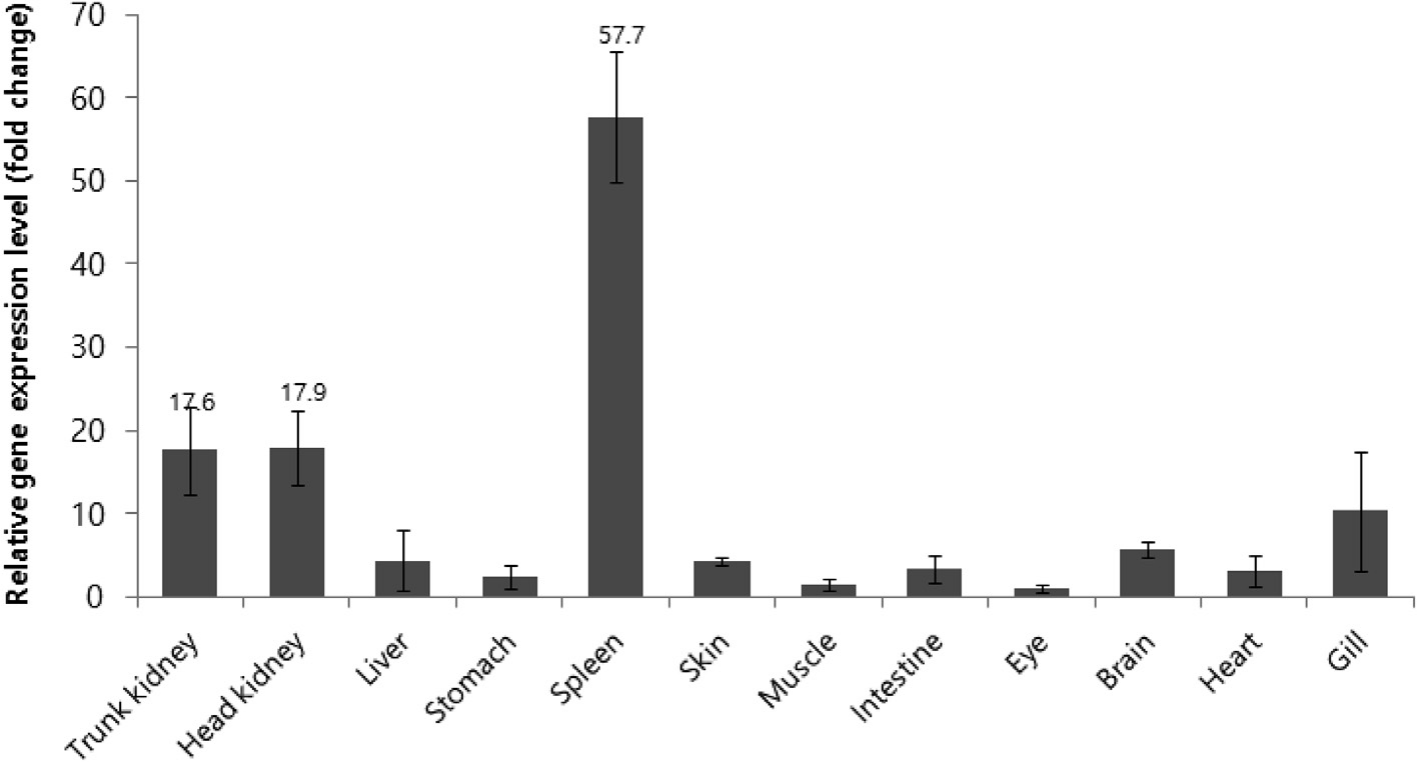
Detection of the CXCL10 genes in different tissues of healthy red seabream by real-time PCR. EF-1α was used for normalizing the real-time PCR results. Data are presented as the mean ± SD from three independent cDNA samples with three replicates from each sample.

**Fig. 5:**
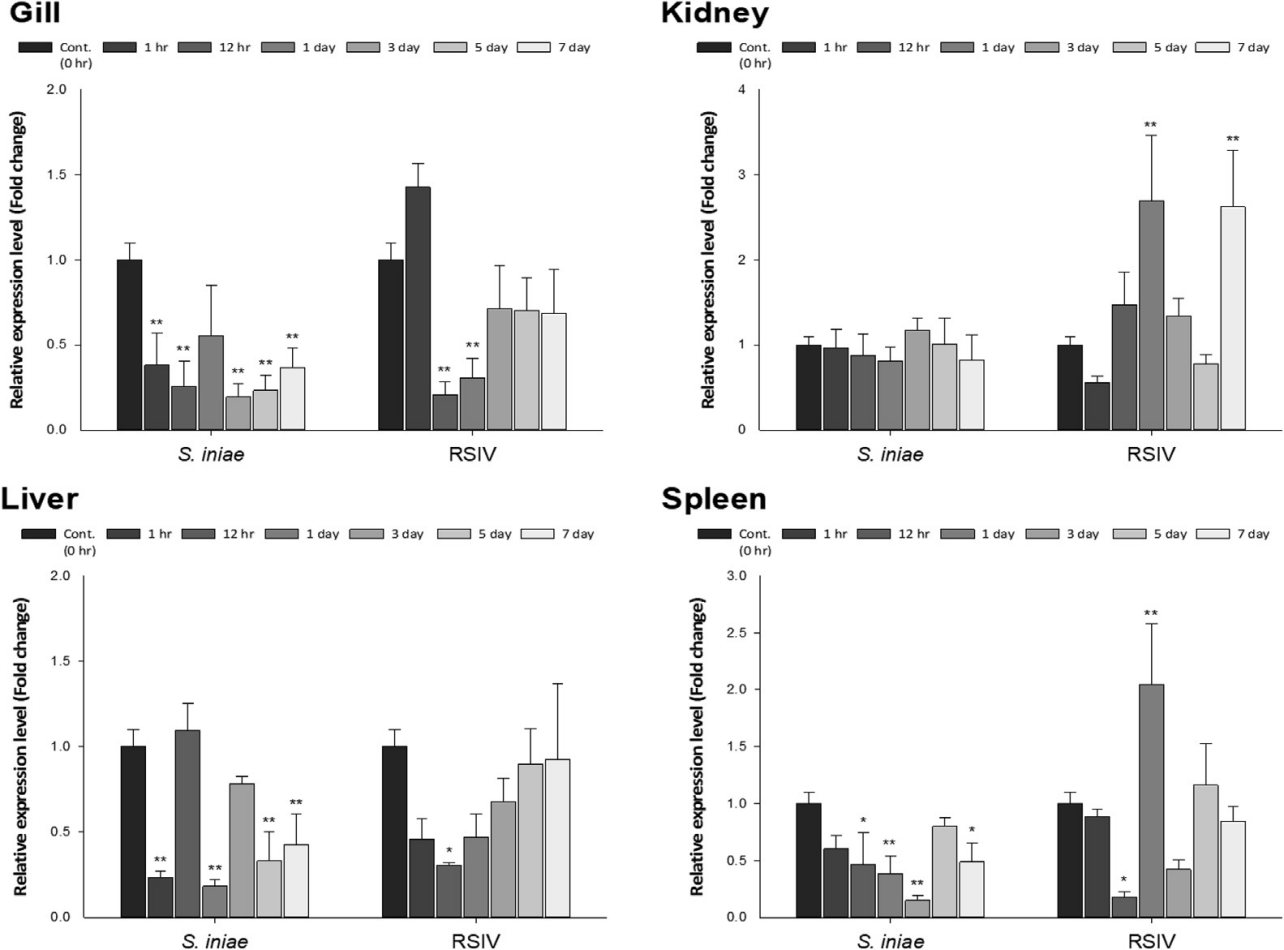
Gene expression of CXCL10 in the gill, kidney, liver and spleen after infection with *S. iniae*, and RSIV. Levels of CXCL10 transcripts were quantified relative to the EF-1α levels. Data are presented as the mean ± SD from three independent cDNA samples with three replicates for each sample. Asterisks represent significant differences compared with the control (PBS) group by ANOVA (*p < 0.05 and **p < 0.01).'

To identify the molecular characteristics of CXCL10, an immunoreactive gene of red seabream, the domain of CXCL10 was identified through sequencing (Fig. 1). Multiple alignment analyses were performed to compare the homology with other fish (Fig. 2), and the phylogenetic tree was analysed by Mega 4 program (Fig. 3), The primer was prepared using Primer 3 (Table 1). Real–time PCR was performed to determine the expression level of CXCL10 in normal and infected tissues (Fig. 4 and Fig. 5).

**Table 1:** Primer sequences used in this study.

Usage	Primer name	Primer sequence (5′–3′)
RT-qPCR (control)	EF-1α (F)	CCTTCAAGTACGCCTGGGTG
	EF-1α (R)	CTGTGTCCAGGGGCATCAAT
RT-qPCR	RsbCXCL10 (F)	GGTGTCTGTGCTCACGTGTC
	RsbCXCL10 (R)	ACTTTCCTCTTGGGGTCCAG

## 2 Experimental design, materials, and methods

### 2.1 Molecular characterization

The nucleotide sequence and the predicted amino acid sequence of the identified full-length CXCL10 cDNA are shown in GENETYX ver. 7.0 program (SDC Software Development, Japan) and the National Centre for Biotechnology Information (NCBI) BLASTX program. The molecular weight (MW) and isoelectric point (p*I*) were predicted using the ProtParam tool of ExPASy Proteomics Serve, and the location of the specific domain was confirmed by Simple Modular Architecture Research Tool (SMART).

The multiple sequence alignment with the amino acid sequence of the antimicrobial peptides of other fish registered in the peptide sequence database of NCBI was analysed using ClustalW. In addition, a phylogenetic analysis was performed using the neighbour-joining (NJ) method of the Mega 4 program, and bootstrap sampling was repeated 2000 times.

### 2.2 Gene expression analysis

#### 2.2.1 Analysis of tissue expression in normal fish

Red seabream was obtained from the Fisheries Resources Research Institute of Gyeongsangnam-do and incubated in a 0.5 t tank at 20–23 °C for 2 weeks. Three red seabreams were anaesthetized with benzocaine (Sigma, USA), and blood cells and tissues were sampled. To separate the peripheral blood leukocytes (PBLs) and red blood cells (RBCs), heparin - treated syringes were used to collect blood from the microarray. The collected blood was added to RPMI 1640 (Invitrogen, USA) to separate the blood cells into 51% Percoll density gradients (Sigma). Percoll: 5 ml of Percoll solution mixed with 10x Phosphate buffer saline (PBS): 1x PBS at a ratio of 51: 9: 40, and 5 ml of the blood suspended in RPMI1640 were divided into layers, and centrifuged for 20 minutes. The separated PBLs and RBCs were washed three times with 1 × PBS, and the pellet obtained from the centrifugation was used for the experiment. After the blood was collected, the red seabream specimens were dissected, and the kidney, head kidney, liver, stomach, spleen, skin, muscle, intestine, eye, brain, heart and gill were extracted from the trunk.

Total RNA was isolated by TRIzol (Invitrogen) by pooling all of the blood cells and extracted tissues from the red sea bream. That is, 500 μl of TRIzol was added to each sample and homogenized with a homogenizer. Then, 100 μl of chloroform (Invitrogen) was added and vortexed, followed by centrifugation at 14,000 rpm for 10 minutes. The supernatant was transferred to a new 1.5 ml tube and equilibrated with PCI (phenol: chloroform: isoamylalcohol) and centrifuged at 14,000 rpm for 10 minutes. Then, the supernatant was transferred to a new 1.5 ml tube, and 500 μl of Isopropanol (Sigma), 5 μl of Dr. Gen (TaKaRa, Japan), and 50 μl of 3 M sodium acetate (TaKaRa) were added, and centrifugation at 14,000 rpm for 10 minutes was performed. After removing the supernatant, 600 μl of 75% DEPC ethyl alcohol was added prior to centrifugation at 14,000 rpm for 5 minutes. Finally, the supernatant was removed and dried naturally at room temperature for 10–15 minutes, followed by the addition of DEPC DDW (30–40 μl).

Before the synthesis of the cDNA, the separated total RNA was DNase treated with RQ1 RNase-free DNase (Promega, USA) according to the manufacturer's method. The treated total RNA was purified using the Transcriptor First Strand cDNA Synthesis Kit (Mannheim, Germany) according to the manufacturer's instructions. One microlitre of DNase-treated total RNA, 1 μl of anchored-oligo (dT) 20 primer, and 11 μl of water were mixed and reacted at 65 °C for 10 minutes and then on ice for 5 minutes. A total of 20 μl of a mixture of Transcriptor Reverse Transcriptase Reaction Buffer (4 μl), Protector RNase Inhibitor (0.5 μl), Deoxynucleotide Mix (2 μl) and Transcriptor Reverse Transcriptase (0.5 μl) were reacted at 55 °C for 30 minutes and 85 °C for 5 minutes.

The specific primer sets used for quantitative real-time PCR were Primer3 ver. 3 based on the full-length sequences of cDNA of CXCL10. (Table 1).

Quantitative real-time PCR was performed using SYBR Green Master Mix (TaKaRa) according to the manufacturer's manual to determine the expression level of CXCL10 in the normal red sea bream. That is, 1 μl of cDNA template, 1 μl of forward and reverse primers, 12.5 μl of SYBR Green, and 9.5 μl of DDW were mixed to a total amount of 25 μl. The amplification conditions were 45 cycles of initial denaturation at 50 °C for 4 minutes and 95 °C for 10 minutes, followed by 95 °C for 20 seconds and 60 °C for 1 minute, final dissociation was performed at 95 °C for 15 seconds, 60 °C for 30 seconds, and 95 °C for 15 seconds. The expression level was compared with the EF-1α mRNA expression level, and 3 repetitions were performed for each gene for the accuracy of experiment. The expression level of each gene was calculated by the 2^-ΔΔCT^ method [1], and all data were expressed as the mean ± SD.

#### 2.2.2 Expression analysis by time after pathogen infection

*S. iniae* and RSIV were suspended in PBS and injected into the peritoneal cavity of red sea bream at 1.5 × 10⁵ cells/fish and 1.5 × 10⁴ copies/fish, respectively [2]. Control group was injected with an equal volume PBS intraperitoneally. Each experimental and control group was housed in a 1.5 ton water tank, and the water temperature was maintained at 23–26 °C. After injection of the pathogen and PBS, 3 individuals were randomly selected from each experimental group and control group on days 1, 3, 5 and 7, and kidney, liver, gill and spleen tissues were extracted. The extracted tissues were stored at −80 °C until use in the experiments. Total RNA isolation and cDNA synthesis were performed in the same manner as described for the tissue expression in normal recipients. To analyse the immune response of CXCL10 to a variety of pathogens, quantitative real-time PCR was performed as described above. The expression level of each gene was calculated by the 2^-ΔΔCT^ method [1] and all data were expressed as the mean ± SD. Significant differences between the groups were confirmed by one-way ANOVA (*p < 0.05 and **p < 0.01), except for values lower than the control.
