# Supplementary material for: Data on CXC chemokine ligand 10(CXCL10) expression and activation in red sea bream during bacterial and viral infection
Source: Data Brief. 2019 Apr 23;25:103943. doi: 10.1016/j.dib.2019.103943 (PMC6626878; doi:10.1016/j.dib.2019.103943)
Supplement: Multimedia component 1 [file mmc1.pdf]

Conflict of Interest and Authorship Conformation Form

Please check the following as appropriate:

- ○ All authors have participated in (a) conception and design, or analysis and interpretation of the data; (b) drafting the article or revising it critically for important intellectual content; and (c) approval of the final version.
- ○ This manuscript has not been submitted to, nor is under review at, another journal or other publishing venue.
- ○ The authors have no affiliation with any organization with a direct or indirect financial interest in the subject matter discussed in the manuscript
- ○ The following authors have affiliations with organizations with direct or indirect financial interest in the subject matter discussed in the manuscript:

Author's name	Author's signature
Won-Sik Woo	
Min Soo Joo	
Jee Youn Hwang	
Mun-Gyeong Kwon	
Jung Soo Seo	
Seong Don Hwang	
Bo-Yeong Jee	
Mu-Chan Kim	
Chan-Il Park	
